# Current COVID-19 treatments: Rapid review of the literature

**DOI:** 10.7189/jogh.11.10003

**Published:** 2021-04-24

**Authors:** Yijia Dong, Azwa Shamsuddin, Harry Campbell, Evropi Theodoratou

**Affiliations:** 1 Edinburgh Medical School, College of Medicine and Veterinary Medicine, The University of Edinburgh, Edinburgh, UK; 2 Centre for Medical Informatics, Usher Institute, The University of Edinburgh, Edinburgh, UK; 3 Centre for Global Health, Usher Institute, The University of Edinburgh, Edinburgh, UK; 4 Cancer Research UK Edinburgh Centre, Medical Research Council Institute of Genetics and Molecular Medicine, University of Edinburgh, Edinburgh, UK

## Abstract

**Background:**

As SARS-CoV-2 continues to spread worldwide, it has already resulted in over 110 million cases and 2.5 million deaths. Currently, there are no effective COVID-19 treatments, although numerous studies are under way. SARS-CoV-2, however, is not the first coronavirus to cause serious outbreaks. COVID-19 can be compared with previous human coronavirus diseases, such as Severe Acute Respiratory Syndrome (SARS) and Middle East Respiratory Syndrome (MERS), to better understand the development of treatments.

**Methods:**

Databases Medline, Embase and WHO COVID-19 was systematically searched on 9 February 2021 for studies reporting on therapeutic effect of COVID-19 treatments. Clinical trials, case reports, observational studies and systematic reviews in the English language were eligible.

**Results:**

1416 studies were identified and 40 studies were included in this review. Therapies included are: remdesivir, convalescent plasma, hydroxychloroquine, lopinavir/ ritonavir, interferon, corticosteroids, cytokine storm inhibitors and monoclonal antibodies. Remdesivir, convalescent plasma and interferon seems to provide some clinical benefits such as faster recovery time and reduced mortality, but these effects are not clinically significant. Some corticosteroids are effective in reducing mortality in severe COVID-19 patients. Hydroxychloroquine do not convey any beneficial, and therapies such as cytokine storm inhibitors and monoclonal antibodies were also not effective and require further investigation.

**Conclusions:**

There is no single therapy effective against COVID-19. However, a combination of therapies administered at different stages of infection may provide some benefit. This conclusion is reflected in the limited effects of these treatments in previous human coronaviruses.

The World Health Organisation (WHO) declared the coronavirus disease (COVID-19) outbreak as a global pandemic on 12 March 2020. As the novel coronavirus SARS-CoV-2 continues to spread worldwide, it has already resulted in over 110 million cases and 2.5 million deaths. Multiple mutant strains have also complicated the landscape. While numerous studies are under way worldwide to uncover its prevention and cure, there are currently no known therapeutics for COVID-19. SARS-CoV-2, however, is not the first coronavirus to cause serious outbreaks. COVID-19 can be compared with previous human coronavirus diseases, such as Severe Acute Respiratory Syndrome (SARS) and Middle East Respiratory Syndrome (MERS), to better understand the development of treatments. This rapid review with elements of evidence briefing aims to provide a comprehensive overview of current and emerging COVID-19 treatment options based on clinical trials. Finally, we will highlight new emerging therapies and the future prospects of this pandemic.

Since its discovery in December 2019, the coronavirus SARS-CoV-2 has been responsible for the worldwide COVID-19 pandemic. The coronavirus infects humans through the binding of its spike glycoprotein (S) with the host cell receptor, angiotensin-converting-enzyme 2 (ACE2). Peak viral replication occurs in the initial 7-10 days of infection and the primary immune response occurs in days 10-14. This progresses to either viral clearance or an uncontrolled immune response (cytokine storm) [1]. The time of therapy administration is crucial in determining COVID-19 outcomes. However, with no known cure, as of 8 March 2021, more than 100 million people have been infected and at least 2.5 million have died [2]. In addition, mutant strains have contributed to further uncertainty. The major impact on human health is only one aspect of the pandemic. Public health interventions to “flatten the curve”, including nationwide lockdowns, social distancing and travel restrictions, will have lasting effects on the economy, human behaviour and education [3]. Due to the absence of effective treatments and vaccines, current measures are largely reliant on public compliance which have uncertain benefits [4] and puts the world at risk of multiple waves of infections [5]. It is therefore of great interest and urgency that treatments for COVID-19 should be found.

There are currently seven coronaviruses known to infect humans: the endemic HCoV-HKU1, HCoV-OC43, HCoV-NL63 and HCoV-229E which cause 15% of the cases of the common cold; and the epidemic SARS-CoV, MERS-CoV and SARS-CoV-2 (**Table 1**). SARS-CoV which caused SARS and MERS-CoV which causes MERS are much more deadly than previous coronaviruses. SARS rapidly spread to 29 countries globally, causing an epidemic with 8096 cases, 774 mortalities and a CFR of 11% [13]. Likewise, MERS spread to more than 27 countries and as of November 2019 has resulted in 2494 cases and 858 deaths. Its 35% CFR makes it the deadliest coronavirus to date [27]. As patients acquire immunopathological damage, they can present with dangerous complications such as acute respiratory distress syndrome (ARDS) which developed in around 16% of SARS [28] and over 50% of MERS patients [18].

**Table 1:** Characteristics of coronaviruses that infect humans

Coronavirus	Date discovered	Dated diverged from common ancestor	Class/ genera	Symptoms	Transmission	Incubation period (days)	Risk Factors
HCoV-229E	1966, Chicago	1800s [6]	Alpha	Common cold symptoms: rhinorrhoea, nasal congestion, sore throat, headache and chills [7]. Occurrences of lower respiratory tract infection (LRTI) mainly in children [8]	Respiratory	2-4 [7]	Immunocompromised, children, elderly.
HCoV-OC43	1967	1850-1900 [9]	Beta	Common cold symptoms. Higher rates of LRTI in adults than HCoV-229E [10]	Respiratory	2-4 [7]	Immunocompromised, children, elderly.
SARS-CoV	2002, Guangdong	1985-1998 [9]	Beta	Fever, dry cough, headache, dizziness, rhinorrhoea, myalgia, chills, rigors, diarrhoea, vomiting. LRTI, acute respiratory distress syndrome (ARDS), shock, multi-organ failure [11]	Mainly Respiratory. Faeces-oral, to a lesser extent [12]	2-7 [13]	Advanced age, male sex [14]. No recorded deaths in children and teenagers [11].
HCoV-NL63	2004, Netherlands	1200s [9]	Alpha	Common cold symptoms [7], LRTI [15]	Respiratory	2-4 [7]	Immunocompromised, children, elderly.
HCoV-HKU1	2005, Hong Kong	1950s [9]	Beta	Common cold symptoms [7], diarrhoea, vomiting [16], childhood febrile seizures [17]	Respiratory	2-4 [7]	Immunocompromised, children, elderly, smoking, inhaled corticosteroids [16].
MERS-CoV	2012, Saudi Arabia	2006 [9]	Beta	Fever, chills, cough, myalgia and gastrointestinal symptoms LRTI, ARDS, multi-organ failure, renal failure [18]	Respiratory. Requires close and prolonged contact [19]	2-14 [20]	Advanced age, male sex, chronic conditions (present in 75% of patients) such as diabetes, obesity, hypertension, lung conditions, cardiac conditions [18,21].
SARS-CoV-2	2019	Not known	Beta	Fever, dry cough, dyspnoea, myalgia and fatigue [22]. Less gastrointestinal involvement than SARS and MERS [23]	Primarily respiratory droplets. To a lesser extent: faecal-ran and through eyes [24]. Airborne transmission during aerosol generating procedures. Airborne transmission in indoor settings with poor ventilation under investigation [25]	5-6 d on average. Up to 14 d [25]	Advanced age, male sex. Co-morbidities (present in 20%-51% of patients): hypertension, diabetes, cardiovascular disease, pulmonary disease and malignancy [26].

ARDS is a set of pulmonary conditions caused by uncontrolled inflammation, leading to acute respiratory failure. In response to SARS-CoV, MERS-CoV and SARS-CoV-2, the body produces a massive immune response, producing pro-inflammatory neutrophils and cytokines like IL-6, TNFα and IL-1β [29]. This “cytokine storm” attacks lung tissue and pneumocytes to cause vascular leakage and pulmonary fibrosis. Damage to type 1 cells cause decreased perfusion by shunting while damage to type 2 cells puts patients at risk of lung collapse due to decreased surfactant production. Thrombi formation due to activation of the coagulation cascade in response to the fluid exudates further restricts oxygen exchange [30].

SARS-CoV-2 is the first coronavirus to cause a pandemic of its scale in modern times, which could be attributed to a high basic reproduction number (R0). The R0 of COVID-19 is estimated to be 3.77, which is higher than both SARS and MERS [31]. After the incubation period, COVID-19 patients develop moderate symptoms lasting around 5-8 days [32]. Around 5%-15% of COVID-19 patients develop ARDS, acute organ failure and shock [33]. The case fatality rate (CFR) varies by country - from less than 0.1% in Singapore to over 19% in Yemen [34]. Chest x-rays appear consistent with that of pneumonia, and laboratory findings include lymphopenia, thrombocytopenia, and leukopenia [22]. Similar to SARS and MERS, children are less likely to become infected and present with milder cases [35] whereas older men with co-morbidities have higher mortality [36].

In face of the current crisis, numerous treatments are being tested based on past experience of SARS and MERS. There are 3 common approaches for the discovery of COVID-19 drugs. The first is screening chemical libraries with databases containing large numbers of existing compounds that may have antiviral properties (eg, remdesivir). Second is the testing of existing broad-spectrum antiviral drugs (eg, ribavirin and interferons). The final method involves the de novo development of novel, specific agents such as monoclonal antibodies based on the genomic and biophysical understanding of the SARS-CoV-2 [37]. Development of novel agents takes years and given the urgency of the current pandemic, COVID-19 treatments are mainly repurposed from SARS and MERS.

Promising treatments have been approved for worldwide clinical trials. Remdesivir; Lopinavir/Ritonavir; Interferon β; hydroxychloroquine and dexamethasone are some of the drugs included in the WHO Solidarity Trial and the Recovery Trial [38,39]. Novel vaccines, emerging “cytokine storm” targeting treatments and convalescent plasma therapies are also being explored. Aiming to provide a comprehensive overview of the area of COVID-19 therapeutics, the main objective of this rapid review is to describe existing SARS-CoV-2 therapeutic approaches and present the results of their current COVID-19 clinical trials.

## METHODS

The databases Medline, Embase and WHO COVID-19 were searched for all publications from 2019 to 9 February 2021 using a combination of the terms “COVID 19”, “SARSCoV2”, “coronavirus”, “therapy”, “lopinavir”, “ritonavir”, “ribavirin”, “remdesivir”, “hydroxychloroquine”, “corticosteroids”, “anakinra”, “tocilizumab”, “convalescent plasma”, “interferon” and “monoclonal antibodies”. (Table S1 in the **Online Supplementary Document**). From the articles retrieved, additional references were identified by a manual search among the cited references and through searching journals online. The types of studies included were clinical trials, case reports, observational studies and systematic reviews. To prevent bias, studies with less than 10 patients were not included. Articles which did not have full text available or were not in English were also excluded.

Data extracted from eligible studies included: Drug/treatment, author, year, study aim, study type, study design, status, main findings and limitations.

The literature search and screening for eligible articles was conducted by 2 reviewers (AS, YD). Data extraction was performed by 2 reviewers (AS, YD).

## RESULTS

**Figure 1:**
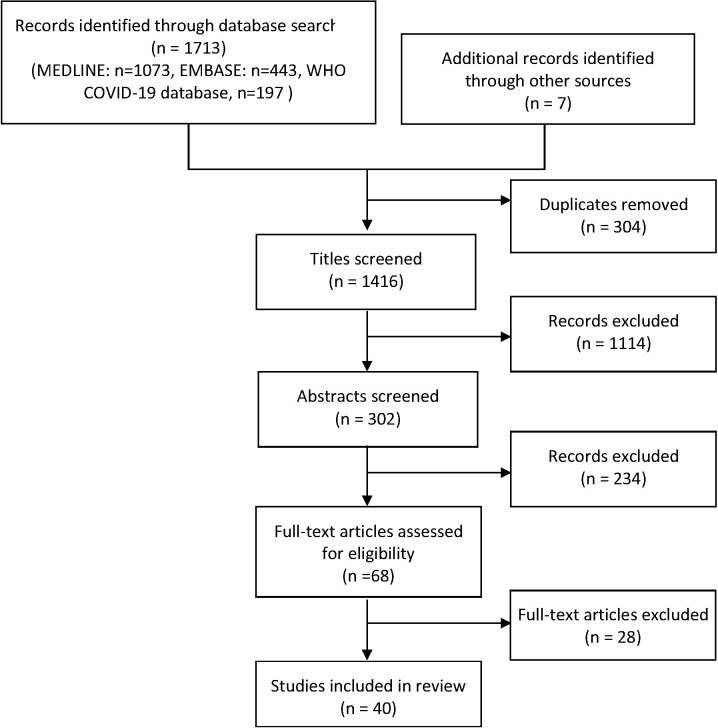
Flowchart summarising study identification and selection.

After removal of duplicates, the literature search identified 1409 unique articles. A further 7 studies were obtained through manual and citation searches. From the 1416 total articles identified, 1114 were excluded after title review, 234 were excluded after abstract review. Sixty-eight articles were reviewed by full text and a total of 40 articles were selected to be included (**Figure 1**). This rapid review includes 4 studies on remdesivir, 6 on convalescent plasma, 7 on hydroxychloroquine (**Table 2**), 2 on lopinavir/ ritonavir, 4 on interferon, 7 on corticosteroids, 6 on cytokine storm inhibitors (**Table 3**) and 6 on monoclonal antibodies (**Table 4**). **Table 2****,** **Table 3** and **Table 4** summarise the studies we reviewed regarding current COVID-19 therapy and trials.

**Table 2:** Summary of COVID-19 therapy studies: Compounds with anti-viral properties

No	Drug/treatment	Author	Year	Study aim	Study type	Study design	Status	Main findings	Limitations
1.	Remdesivir	Grein et al [40].	2020	To describe outcomes in a cohort of patients hospitalised for severe COVID-19 who were treated with remdesivir on a compassionate-use basis	Open label program.	Compassionate use of remdesivir approved by manufacturer for hospitalised COVID-19 patients with RT-PCR confirmed infection and needing oxygen support. 61 patients received remdesivir treatment; 8 excluded due to missing data and findings of 53 patients reported.	Complete	Clinical improvement was observed in 36 of 53 patients (68%). Measurement of efficacy will require ongoing randomised, placebo-controlled trials of remdesivir therapy.	Short follow-up (median 18 d, interquartile range [IQR] = 13-23), small cohort, no control group. Study funded by manufacturers of remdesivir.
		Wang Y. et al [41].	2020	To assess the effectiveness and safety of intravenous remdesivir in adults admitted to hospital with severe COVID-19.	Double-blinded, multicentre, placebo-controlled RCT.	237 patients admitted to 10 hospitals in Wuhan with laboratory-confirmed COVID-19 and classified as severe were randomly assigned in a 2:1 ratio to remdesivir (n = 158) or placebo (n = 79) groups.	Complete	Remdesivir was not associated with statistically significant clinical benefits (hazard ratio [HR] = 1.23 95% confidence interval [CI] = 0.87-1.75]), yet there was a numerical reduction in time to clinical improvement in those treated earlier with remdesivir which requires confirmation in larger studies.	Failed to complete full enrolment (owing to the end of the outbreak), insufficient power to detect assumed differences in clinical outcomes, initiation of treatment quite late in COVID-19, and the absence of data on infectious virus recovery or on possible emergence of reduced susceptibility to remdesivir. One author served as a non-compensated consultant to manufacturers of remdesivir.
		Beigel et al [42]	2020	To evaluate the clinical efficacy and safety of remdesivir among hospitalised adults with laboratory-confirmed COVID-19.	Double-blinded, multicentre, placebo-controlled RCT.	1062 hospitalised patients across 60 international trial sites randomly assigned in a 1:1 ratio to remdesivir (n = 538) or placebo (n = 521) groups.	Complete	Remdesivir was superior to placebo in shortening the time to recovery (relative risk [RR] 1.29 95% CI = 1.12-1.49) in adults hospitalised with COVID-19 and evidence of lower respiratory tract infection.	Report based on preliminary findings. Primary outcome changed early in the trial.
		Spinner et al [43]	2020	To evaluate the efficacy and adverse events of remdesivir administered for 5 or 10 day (d) vs standard care in hospitalized patients with moderate COVID-19	Randomised, open label, multicentre trial	584 hospitalised patients across 105 international sites randomly assigned in a 1:1:1 ratio to receive up to a 5-d course of remdesivir (n = 191) up to a 10-d course of remdesivir (n = 193), or standard care (n = 200).	Complete	On day 11, patients in the 5-d remdesivir group had statistically significantly higher odds of a better clinical status distribution than those receiving standard care (odds ratio [OR] = 1.65 95% CI = 1.09-2.48; *P* = 0.02). The clinical status distribution on day 11 between the 10-d remdesivir and standard care groups was not significantly different (*P* = 0.18)	Based on preliminary data leading to change in protocol, open-label design may have led to biases. Effects on viral load not assessed and decisions on discharge may have been driven by factors other than clinical improvement.
2.	Convalescent plasma	Salazar et al [44]	2020	To provide additional data on initial clinical observations of patients’ clinical course and subsequent improvement after receiving convalescent plasma therapy for COVID-19	Therapy study.	25 patients hospitalised with severe/critical COVID-19 transfused with Convalescent-Phase Donor Plasma using either the emergency investigational new drug (eIND) or investigational new drug (IND) applications approved by the US FDA.	Complete	At day 7 post-transfusion with convalescent plasma, 9 patients had at least a 1-point improvement in clinical scale, and 7 of those were discharged. By day 14 post-transfusion, 19 (76%) patients had at least a 1-point improvement in clinical status and 11 were discharged. No adverse events as a result of plasma transfusion was observed.	Small case series with no control group. All patients received adjunct therapy (azithromycin, ribavirin, remdesivir. etc.) as patients were critically ill.
		Abolghasemi et al [45]	2020	To explore the efficacy of administrating convalescent plasma to COVID-19 patients in a nonrandomized multi-centre clinical trial.	Multicentre clinical trial	189 hospitalised patients across 6 sites were assigned to convalescent plasma group (n = 115) or control group (n = 74)	Complete	98 (98.2%) of patients who received convalescent plasma were discharged from hospital compared to 56 (78.7%) patients in control group. Length of hospitalization days was significantly lower (9.54 d) in convalescent plasma group com- pared with that of control group (12.88 d). Only 8 patients (7%) in convalescent plasma group required intubation while that was 20% in control group.	Not randomised. This led to bias of clinicians and the control group was smaller and consisted of milder patients. Standard therapy allowed in both groups and not standardized.
		Alsharidah et al [46]	2021	To assess the effectiveness of CCP in both moderate and severe COVID-19 cases compared to the standard treatment alone.	Multicentre, prospective cohort study	135 hospitalised patients across 4 sites with moderate to severe COVID-19 were enrolled and compared to 233 patients receiving standard care.	Complete	Among those with moderate COVID-19 disease, time to clinical improvement was 7 d in the CCP group, vs 8 d in the control group (*P* = 0.006). 30-d mortality rate was also significantly lower. For severe COVID-19 disease, time to clinical improvement was 7 d in the CCP group, vs 15.5 d in the control group (*P* = 0.003). There were no serious adverse effects.	Lack of randomisation, clinical treatment and management not standardised, did not exclude donors who were negative for IgG antibodies.
		Li L. et al [47]	2020	To evaluate the efficacy and adverse effects of convalescent plasma added to standard treatment, compared with standard treatment alone, for patients with severe or life-threatening COVID-19.	Multicentre, open label, randomised clinical trial.	103 patients with laboratory-confirmed COVID-19 and classified as severe/critical, randomly assigned to convalescent plasma in addition to standard treatment (n = 52) vs standard treatment alone (control) (n = 51), stratified by disease severity.	Complete	Clinical improvement occurred within 28 d in 51.9% (27/52) of the convalescent plasma group vs 43.1% (22/51) in the control group (difference, 8.8% 95% CI = -10.4% to 28.0%; HR = 1.40, 95% CI = 0.79-2.49). There was no significant difference in 28-d mortality (15.7% vs 24.0%; OR = 0.59, 95% CI = 0.22-1.59]) or time from randomization to discharge (51.0% vs 36.0% discharged by day 28: HR = 1.61 95% CI = 0.88-2.95]). Two patients in the convalescent plasma group experienced adverse events within hours after transfusion that improved with supportive care.	Small sample size and study terminated early due to lack of new cases emerging in Wuhan. Possibility for study to be underpowered to detect a clinically important benefit of convalescent plasma therapy. Median time between the onset of symptoms and randomization was 30 d. This was an open-label study, the primary outcome was based to some degree on physicians’ clinical management decisions. Standard therapy allowed in both groups and not protocolized.
		Agarwal et al [48]	2020	To investigate the effectiveness and safety of convalescent plasma in patients with moderate COVID-19 admitted to hospitals across India to limit progression to severe disease	Open label phase 2 multicentre RCT	464 patients across 39 sites in India randomly assigned 1:1 to receive convalescent plasma (n = 235) or standard care (n = 229)	Complete	Progression to severe disease or all-cause mortality at 28 d after enrolment occurred in 44 (19%) participants in the intervention arm and 41 (18%) in the control arm (risk difference 0.008, 95% CI = −0.062 to 0.078); RR = 1.04, 95% CI = 0.71 to 1.54). No difference in inflammatory markers.	Open label susceptible to anchoring bias, testing for biomarkers were from different manufacturers, bias in enrolment numbers between sites due to pandemic at different stages across India.
		Zeng Q.L et al [49]	2020	To retrospectively collect and analyse data of patients who received and did not receive convalescent plasma therapy to evaluate its efficacy.	Retrospective, observational study.	Extracted the epidemiological, demographic, clinical, laboratory, management, and outcome data of 21 COVID-19 patients who received (n = 6) and did not receive (n = 15) convalescent plasma.	Complete	All 6 critically ill patients who received plasma transfusion at a median of 21.5 d after first detection of viral shedding tested negative for SARS-CoV-2 RNA 3 d after infusion, yet 5 died eventually. Convalescent plasma treatment can discontinue SARS-CoV-2 shedding but cannot reduce mortality in critically end-stage COVID-19 patients, and treatment should be initiated earlier.	Limited number of patients due to end of outbreak in Wuhan. The amount of viral antibodies given to each patient was unknown and not standardized, which may lead to different clinical outcomes.
3.	Hydroxychloroquine (HCQ)/chloroquine (CQ)	Mahévas et al [50]	2020	To assess the effectiveness of hydroxychloroquine in patients admitted to hospital with coronavirus disease 2019 (COVID-19) pneumonia who require oxygen.	Observational comparative study.	Data of 181 patients with severe COVID-19 who required oxygen split into those who did receive hydroxychloroquine (n = 92) and those who did not (n = 89). Analysed for 21 d survival.	Ongoing	The survival rate without transfer to the intensive care unit at day 21 was 76% in the treatment group and 75% in the control group (weighted HR = 0.9, 95% CI = 0.4-2.1). Overall survival at day 21 was 89% in the treatment group and 91% in the control group (HR = 1.2, CI = 0.4. to 3.3).	Not randomised causing potential for bias, prognostic variables not balanced, small sample.
		Gautret et al [51]	2020	To evaluate the effect of HCQ on respiratory viral loads.	Clinical trial	36 hospitalised patients with laboratory-confirmed COVID-19 in multiple hospitals served as either the treatment group (n = 26) or control (n = 16).	Ongoing	Six patients were asymptomatic, 22 had upper respiratory tract infection symptoms and 8 had lower respiratory tract infection symptoms. Twenty cases were treated in this study (6 lost to follow-up) and showed a significant reduction of the viral carriage at D6-post inclusion compared to controls, and much lower average carrying duration than reported in the literature for untreated patients. Azithromycin added to HCQ was significantly more efficient for virus elimination (*P* < 0.001).	Based on preliminary findings. Study had a small sample size, limited long-term outcome follow- up, and dropout of 6 patients from the study.
		The RECOVERY Collaborative Group [52]	2020	To report the results of a comparison between hydroxychloroquine and usual care involving patients hospitalized with COVID-19.	Open-label RCT	4716 patients from 176 UK sites from randomly assigned to receive hydroxychloroquine (n = 1561) or standard care (n = 3155).	Complete	Death within 28 d occurred in 421 patients (27.0%) in the hydroxychloroquine group and in 790 (25.0%) in the usual-care group (RR = 1.09; 95% CI = 0.97-1.23; *P* = 0.15). patients in the hydroxychloroquine group were less likely to be discharged from the hospital alive within 28 d than those in the usual-care group (59.6% vs 62.9%; RR = 0.90; 95% CI = 0.83-0.98)	Does not investigate use as prophylaxis or in patients with less severe infection.
		Borba et al [53]	2020	To evaluate the safety and efficacy of 2 CQ dosages in patients with severe COVID-19.	Parallel, double-masked, randomised, phase 2b clinical trial	81 patients hospitalised with severe COVID-19 randomised at a 1:1 ratio into high dosage (n = 41) or low dosage (n = 40) groups.	Ongoing	Viral RNA was detected in 31 of 40 (77.5%) and 31 of 41 (75.6%) patients in the low dosage and high dosage groups, respectively. Lethality until day 13 was 39.0% in the high dosage group (16 of 41) and 15.0% in the low dosage group (6 of 40). The high dosage group presented more instance of QTc interval greater than 500 milliseconds (7 of 37, 18.9%) compared with the low-dosage group (4 of 36, 11.1%). Respiratory secretion at day 4 was negative in only 6 of 27 patients (22.2%).	Patients enrolled before laboratory confirmation of COVID-19 diagnosis. Based on preliminary findings. Small sample size, single centre, and lack a placebo control group.
		Boulware et al [54]	2020	To evaluate postexposure prophylaxis with HCQ after exposure to COVID-19.	Randomized, double-blind, placebo-controlled clinical trial.	821 asymptomatic adult participants with known exposure to a person with laboratory-confirmed COVID-19 randomised into HCQ group (n = 414) or placebo group (n = 407).	Complete	The incidence of new illness compatible with COVID-19 did not differ significantly between participants receiving HCQ (49 of 414, 11.8%) and those receiving placebo (58 of 407, 14.3%); the absolute difference was -2.4 percentage points (95% CI = 7.0-2.2; *P* = 0.35). Side effects were more common with HCQ than with placebo (40.1% vs 16.8%).	An a priori symptomatic case definition was used to define probable COVID-19 as diagnostic testing was lacking for vast majority of participants. Data obtained through participant self-report.
		Huang et al [55]	2020	To evaluate the efficacy and safety of CQ in hospitalized patients with COVID-19.	RCT	22 hospitalised patients with RT-PCR confirmed COVID-19 randomly assigned into treatment with CQ (n = 10) and treatment with Lopinavir/Ritonavir, which served as a control group (n = 12).	Ongoing	At day 14, all 10 patients (100%) from the CQ group were discharged compared to 6 patients (50%) from the Lopinavir/ Ritonavir group. The incidence rate of lung improvement based on CT imaging from the CQ group was more than double to that of the Lopinavir/Ritonavir group (RR 2.21, 95% CI = 0.81-6.62). 5 patients in the CQ group experienced a total of 9 adverse events.	Based on preliminary findings. Small sample size.
		Tang et al [56]	2020	To assess the efficacy and safety of HCQ plus standard of care compared with standard of care alone in adults with COVID-19.	Multicentre, open label, randomised controlled trial.	150 hospitalised patients with laboratory-confirmed COVID-19 randomised to HCQ plus standard of care (n = 75) and standard of care alone (n = 75) groups.	Complete	Administration of HCQ did not result in a significantly higher probability of negative conversion than standard of care alone (difference between groups was 4.1% 95% CI = 10.3%-18.5%) in patients admitted to hospital with mainly persistent mild to moderate COVID-19. Adverse events were higher in HCQ recipients than in non-recipients.	Possibility of biased investigator determined assessments and unbalanced dosage adjustment. Cannot provide evidence on the effect of HCQ on disease progression or regression because 148/150 (99%) patients in trial had mild to moderate disease.

**Table 3:** Summary of COVID-19 therapy studies: Existing broad-spectrum antiviral drugs

No	Drug/treatment	Author	Year	Study aim	Study type	Study design	Status	Main findings	Limitations
1.	Lopinavir, ritonavir, ribavirin	Cao et al [57].	2020	To evaluate the efficacy and safety of oral lopinavir–ritonavir for SARS-CoV-2 infection	Randomised, controlled, open label clinical trial.	199 laboratory-confirmed COVID-19 patients randomised at a 1:1 ratio into lopinavir-ritonavir in addition to standard care (n = 99) and standard care alone (n = 100) groups.	Ongoing	Treatment with lopinavir–ritonavir was not associated with a difference from standard care in the time to clinical improvement (HR = 1.31, 95% CI = 0.95-1.80]). Mortality at 28 d was similar between the groups (19.2% vs 25.0%; difference, -5.8 percentage points; 95% CI = 17.3-5.7).	Based on preliminary data. Lopinavir–ritonavir treatment was stopped early in 13 patients (13.8%) because of adverse events. Possible that knowledge of the treatment assignment might have influenced clinical decision-making.
		Horby et al [58]	2020	To report the results of a randomised trial to assess whether lopinavir– ritonavir improves clinical outcomes in patients admitted to hospital with COVID-19	Open-label, platform RCT	5040 patients from 176 UK sites from randomly assigned 1:2 to receive lopinavir-ritonavir plus standard care (400mg and 100mg) (n = 1616) or standard care alone (n = 3424).	Complete	Treatment does not improve clinical outcome. 374 (23%) of lopinavir–ritonavir patients and 767 (22%) usual care patients died within 28 d (RR 1.03, 95% CI = 0.91-1.17; *P* = 0.60). No significant difference in time until discharge alive from hospital (median 11 d [IQR 5 to >28] in both groups) or the proportion of patients discharged from hospital alive within 28 d (RR = 0.98, 95% CI = 0.91-1.05; *P* = 0.53)	No information collected on non-serious adverse effects or biomarkers. Few intubated patients included so unable to access effectiveness on critical patients.
2.	Interferon (IFN)	Davoudi-Monfared et al [59]	2020	To evaluate the efficacy and safety of IFN-β 1a in patients with severe COVID-19	Randomised clinical trial	81 patients randomised to treatment with IFN-β 1a (n = 42) or control (n = 39).	Complete	Time to the clinical response was not significantly different between the IFN and the control groups (*P* = 0.95). On day 14, 66.7% vs 43.6% of patients in the IFN group and the control group were discharged (OR = 2.5; 95% CI = 1.05 to 6.37). The 28-d overall mortality was significantly lower in the IFN than the control group (19% vs 43.6%, respectively, *P* = 0.015).	Some COVID-19 cases were not confirmed by PCR, patients’ stage of disease not accurately classified.
		Monk et al [60]	2021	To determine whether inhaled SNG001 has the potential to reduce the severity of lower respiratory tract illness and accelerate recovery in patients diagnosed with COVID-19.	Phase 2, double-blind, placebo-controlled, RCT.	98 patients from 9 UK sites randomly assigned 1:1 to the treatment group (n = 48) and placebo (n = 50). Treatment was administered by inhalation for 14 d.	Ongoing	Patients receiving SNG001 had greater odds of improvement (OR = 2.32 95% CI = 1-07-5.04]; *P* = · 033) on day 15 or 16 and were more likely than those receiving placebo to recover to an OSCI score of 1 (no limitation of activities) during treatment (HR 2.19. 95% CI = 1.03-4 = 69; *P* = · 043). SNG001 was well tolerated.	Pilot study, limited sample size, nebuliser unsuitable for patients requiring ventilation. 6 patients withdrew from treatment group and 5 from placebo group.
		Rahmani et al [61]	2020	To assess the efficacy and safety of IFN β-1b in the treatment of patients with severe COVID-19	Open-label, randomised clinical trial	66 patients from one site were randomised at a 1:1 ratio into the treatment (n = 33) and the control group (n = 33) for 2 weeks.	Complete	Time to clinical improvement in the IFN group was significantly shorter than the control group (9 d vs 11 d respectively, *P* = 0.002, HR = 2.30; 95% CI = 1.33–3.39]). At day 14, there was a lower percentage of discharged patients (78.79% vs 54.55) (OR = 3.09; 95% CI = 1.05-9.11, *P* = 0.03). ICU admission rate in the control group was significantly higher than the IFN group (66.66% vs 42.42%, *P* = 0.04 All-cause 28-d mortality was 6.06% and 18.18% in the IFN and control groups respectively (*P* = 0.12).	The effect of IFN on viral clearance was not determined. Small sample size.
		Hung et al [62].	2020	To assess the efficacy and safety of combined interferon beta-1b, lopinavir–ritonavir, and ribavirin for treating patients with COVID-19.	Multicentre, prospective, open label, randomised, phase 2 trial.	127 patients with RT-PCR confirmed COVID-19 randomised at a 2:1 ratio to treatment with combination of lopinavir, ritonavir, ribavirin and IFN (n = 86) and lopinavir and ritonavir (n = 41) groups.	Complete	The combination group had a significantly shorter median time from start of study treatment to negative nasopharyngeal swab (7 d, IQR 5–11) than the control group (12 d, IQR = 8-15; HR = 4.37, 95% CI = 1.86-10.24). Adverse events included self-limited nausea and diarrhoea with no difference between the two groups.	Trial was open label, without a placebo group, and confounded by a subgroup omitting IFN beta-1b within the combination group, depending on time from symptom onset. Study did not include critically ill patients.
3.	Corticosteroids	Zhang et al [36].	2020	To study the epidemiology, clinical features, and short-term outcomes of patients with COVID-19 in Wuhan, China.	Single centre, retrospective, case series study.	Data of 221 laboratory-confirmed COVID-19 patients were analysed for epidemiological, clinical, laboratory and radiological features, treatments, and outcomes.	Complete	A total of 64 (49.6%) patients were given glucocorticoid treatment. The severely affected patients receiving antiviral therapy:50 (90.0%) vs 146 (88.0%); *P* < 0.001) and glucocorticoid treatment: 40 (72.7%) vs 75 (45.2%); *P* < 0.001) were significantly higher than those patients who were not severely affected.	Most patients remain hospitalised.
		Liu Y. et al [63].	2020	To describe the clinical features, treatment, and mortality according to the severity of ARDS in COVID-19 patients.	Single centre, retrospective, cohort study.	Data of 109 laboratory-confirmed COVID-19 patients were analysed for differences in the treatment and progression with the time and severity of ARDS.	Complete	Patients with moderate to severe ARDS were the most likely to receive glucocorticoid therapy (*P* = 0.02) and high-flow nasal oxygen ventilation (*P* < 0.001). No significant effect of antivirus, glucocorticoid, or immunoglobulin treatment was found on survival in COVID-19 patients with ARDS (all log-rank tests *P* > 0.05).	Retrospective study, possibility for systematic selection bias.
		Liu T. et al [64].	2020	To explore changes of markers in peripheral blood of severe COVID-19 patients.	Single centre, retrospective, cohort study.	Data of 69 patients with severe COVID-19 were analysed for clinical characteristics and laboratory examination. 11 non-severe COVID-19 patients were included for comparison.	Complete	The higher level of IL-6 related to glucocorticoids (correlation coefficient [r] = 0.301, *P* = 0.001), human immunoglobulin (r = 0.147, *P* = 0.118), high flow oxygen inhalation (r = 0.251, *P* = 0.007), ventilator therapy (r = 0.223, *P* = 0.017).	Small sample size, retrospective study.
		Zhou et al [65].	2020	To explore risk factors of in-hospital death for patients and describe the clinical course of symptoms, viral shedding, and temporal changes of laboratory findings during hospitalisation.	Multi centre, retrospective, cohort study.	Data of 191 patients with laboratory-confirmed COVID-19 were analysed	Complete	Systematic corticosteroid and intravenous immunoglobulin use differed significantly (*P* = 0.0005) between non-survivors (n = 26; 48%) and survivors (n = 31; 23%).	Some laboratory tests not done in patients, underestimating effects on mortality. Small sample size.
		Tobaiqy et al [66].	2020	To retrospectively evaluate the therapeutic management received by patients with COVID-19 since emergence of the virus.	Systematic review.	41 studies (total 8806 patients) included in review after searching databases Embase, MEDLINE, and Google Scholar.	Complete	Corticosteroid treatment was reported most frequently (n = 25), despite safety alerts issued by WHO and CDC, followed by lopinavir (n = 21) and oseltamivir (n = 16).	Most studies included in the review were of low quality, with incomplete or inconsistent information on study design and outcome.
		WHO REACT working group [67].	2020	To evaluate the 28-d mortality associated with administration of corticosteroids compared with usual care.	Meta-analysis review.	Data of 1703 critically ill patients were pooled from 7 randomized clinical trials that evaluated the efficacy of corticosteroids.	Complete	Administration of dexamethasone (OR = 0.64) and hydrocortisone (OR = 0.69) for critically ill patients lowered the 28-d mortality rate.	The primary meta-analysis was weighted heavily by the RECOVERY trial (57% contribution). One of the studies included may have been subject to bias.
		RECOVERY collaborative group [68].	2021	To report the results of the RECOVERY trial of dexamethasone in hospitalised COVID-19 patients.	Open label RCT.	6425 patients randomised with a 2:1 ratio to dexamethasone (n = 2104) and usual care (n = 4321) groups.	Complete	482 patients (22.9%) in the dexamethasone group and 1110 patients (25.7%) in the usual care group died within 28 d after randomization (age-adjusted RR = 0.83; 95% CI = 0.75–0.93; *P* < 0.001).	Based on early findings.
4.	Drugs targeting the cytokine storm	Cantini et al [69].	2020	To evaluate the clinical impact and safety of Baricitinib therapy for patients with COVID-19.	Pilot study	24 consecutive patients with moderate symptoms were assigned at a 1:1 ratio to baricitinib with ritonavir-lopinavir (n = 12) and control based on ritonavir-lopinavir with hydroxychloroquine (n = 12).	Ongoing	Discharge at week 2 occurred in 58% (7/12) of the baricitinib-treated patients vs 8% (1/12) of controls (*P* = 0.027). At discharge, 57% (4/7) had negative viral nasal/oral swabs.	Pilot study based on early findings. Open label design. No randomisation. Lack of a proper control.
		Bronte et al [70]	2020	Investigate whether baricitinib-induced changes in the immune landscape are associated with a favourable clinical outcome for patients with COVID-19–related pneumonia.	Observational, longitudinal trial.	Of 86 hospitalised patients with COVID-19 related pneumonia, 20 patients received treatment while 56 patients were considered the control	Complete	patients treated with baricitinib had a marked reduction in serum levels of IL-6, IL-1β, and TNF-α, a rapid recovery of circulating T and B cell frequencies, and increased antibody production against the SARS-CoV-2 spike protein	Missing data for some outcomes, short follow-up time, not double-blinded, insufficient evidence to show immune-suppressive features.
		Cao Y. et al [71].	2020	To evaluate the efficacy and safety of ruxolitinib, a JAK1/2 inhibitor, for patients with COVID-19.	Multicentre, prospective, single-blind phase 2 RCT.	43 COVID-19 patients randomised at a 1:1 ratio into ruxolitinib plus standard-of-care (n = 22) and placebo based on standard-of-care treatment (n = 21) groups.	Ongoing	Treatment with ruxolitinib plus standard-of-care was not associated with significantly accelerated clinical improvement in COVID-19 patients (12 (IQR, 10-19) days vs 15 (IQR, 10-18) days; log-rank test *P* = 0.147; HR = 1.669; 95% CI = 0.836-3.335), although ruxolitinib recipients had a numerically faster clinical improvement.	Based on early findings. Small sample size. Patients insisted on nasal cannula oxygen until discharge, which may contribute to the non-statistically significant P value of clinical improvement.
		Roschewski et al [72].	2020	To reduce inflammation and improve clinical outcome of patients with severe COVID-19 by administering acalabrutinib, a highly specific inhibitor of Bruton tyrosine kinase (BTK) for the treatment of lymphoid malignancies.	Prospective, off-label clinical study.	19 hospitalised patients with confirmed COVID-19 and evidence of inflammation and/or severe lymphopenia.	Complete	Among 11 patients in the supplemental oxygen cohort, the median duration of follow-up from the initiation of acalabrutinib treatment was 12 (range, 10 to 14) days. All but one patient received at least 10 d of acalabrutinib, which was the anticipated treatment duration. At the time of formal data collection, eight (73%) patients no longer required supplemental oxygen and had been discharged from the hospital. Among 3 patients still requiring oxygen, one was on 4 L/min by nasal cannula and one was on a ventilator, both with decreasing oxygen requirements, Findings suggest BTK is a likely instigator for the pathological inflammatory response in severe COVID-19.	Findings based on an initial clinical study which has led to a confirmatory international prospective RCT.
		Huet et al [73].	2020	To assess the off-label use of anakinra in patients who were admitted to hospital for severe forms of COVID-19 with symptoms indicative of worsening respiratory function.	Retrospective cohort study.	52 consecutive patients were included in the anakinra group and 44 historical patients were identified in the Groupe Hospitalier Paris Saint-Joseph COVID cohort study for comparison.	Complete	Admission to ICU for invasive mechanical ventilation or death occurred in 13 (25%) patients in the anakinra group and 32 (73%) patients in the historical group (HR = 0.22, 95% CI = 0.11-0.41; *P* < 0.0001). The treatment effect of anakinra remained significant in the multivariate analysis (HR = 0.22, 95% CI = 0.10-0.49]; *P* = 0.0002). An increase in liver aminotransferases occurred in 7 (13%) patients in the anakinra group and 4 (9%) patients in the historical group.	The historical group differed sizeably from the anakinra group for several potentially confounding variables. Obesity was more frequent in the historical group and might have worsened the effects of SARS-CoV-2. In the multivariate analysis of the data, this comorbidity, as well as other between-group differences, did not affect the estimated effect of anakinra on the outcome
		Balkhair et al [74]	2020	To evaluate the efficacy of anakinra in patients who were admitted to hospital for severe COVID-19 pneumonia requiring oxygen therapy.	Prospective, open-label, interventional study	Data was collected from 69 patients with severe COVID-19 pneumonia treated with either anakinra (n = 45) or from a historical control group (n = 24)	Complete	A need for mechanical ventilation occurred in 14 (31%) of the anakinra-treated group and 18 (75%) of the historical cohort (*P* < 0.001). In-hospital death occurred in 13 (29%) of the anakinra-treated group and 11 (46%) of the historical cohort (*P* = 0.082). Patients who received anakinra showed a significant reduction in inflammatory biomarkers.	Small sample size, lack of randomization could have caused bias, controlled group had non standardised treatment, leading to many confounding variables.

**Table 4:** Summary of COVID-19 therapy studies: Novel specific treatment agents

No	Drug/treatment	Author	Year	Study aim	Study type	Study design	Status	Main findings	Limitations
1.	Monoclonal and polyclonal antibodies	Salma et al [75]	2021	TO investigate the safety and efficacy of tocilizumab in hospitalized patients with COVID-19 pneumonia who were not receiving mechanical ventilation.	Randomised, double-blind, placebo-controlled, phase 3 trial	389 patients were randomised at a 2:1 ratio to the treatment group (n = 249) and the placebo group(n = 128).	Complete	patients who had received mechanical ventilation or who had died by day 28 was 12.0% (95% CI, 8.5 to 16.9) in the tocilizumab group and 19.3% (95% CI = 13.3 to 27.4) in the placebo group (hazard ratio for mechanical ventilation or death, 0.56; 95% CI = 0.33 to 0.97; *P* = 0.04 by the log-rank test). Clinical	Treatment in control group was not standardised. Based on preliminary data.
		Hermine et al [76]	2021	To determine whether tocilizumab (TCZ) improves outcomes of patients hospitalized with moderate-to-severe COVID-19 pneumonia	Cohort-embedded, multicentre, open-label, Bayesian randomized clinical trial investigating	130 patients from 9 French hospitals were randomly assigned to the TCZ group (n = 63) or the control (n = 67). Followed up after 28 d.	Complete	At day 14, 12% (95% CI = -28% to 4%) fewer patients needed non-invasive ventilation (NIV) or mechanical ventilation (MV) or died in the TCZ group than in the UC group (24%vs 36%, median posterior hazard ratio HR 0.58; 90% credible interval CrI = 0.33-1.00). No difference in 28 d mortality was found.	Not blinded, lack of standardisation of control group treatment, small sample size, results not generalizable. Preliminary results.
		Veiga et al [77]	2021	To determine whether tocilizumab improves clinical outcomes for patients with severe or critical coronavirus disease 2019	Open label RCT	129 hospitalised patients from 9 sites in Brazil were randomised in a 1:1 ratio to treatment group (n = 65) or standard care group (n = 64).	Complete	Treatment was associated with worse outcomes. 18 of 65 (28%) patients in the tocilizumab group and 13 of 64 (20%) in the standard care group were receiving mechanical ventilation or died at day 15 (OR = 1.54, 95% CI = 0.66 to 3.66; *P* = 0.32). Death at 15 d occurred in 11 (17%) patients in the tocilizumab group compared with 2 (3%) in the standard care group	Open label trial may be subject to bias. Reduction in statistical power due to small sample size and incompatible seven level ordinal scale with proportional odds assumptions. Trial prematurely interrupted due to high death rate.
		Weinreich et al [78]	2021	To describe the initial results involving 275 symptomatic patients from the ongoing phase 1-3 trial involving outpatients with confirmed SARS-CoV-2 infection	Double-blind, phase 1-3 trial, placebo-controlled, RCT	275 un-hospitalised patients with COVID-19 randomly assigned in a 1:1:1 ratio to receive placebo (n = 93), high dose REGN-COV2 (n = 90) or low dose REGN-COV2 (n = 92).	Ongoing	The REGN-COV2 antibody cocktail reduced viral load, with a greater effect in patients whose immune response had not yet been initiated or who had a high viral load at baseline. 6% of the patients in the placebo group and 3% of the patients in the combined REGN-COV2 dose groups reported at least one medically attended visit.	No formal hypothesis testing was performed to control type 1 error. Analyses according to baseline viral load were post hoc.

### Compounds with anti-viral properties

#### Remdesivir

**Figure 2:**
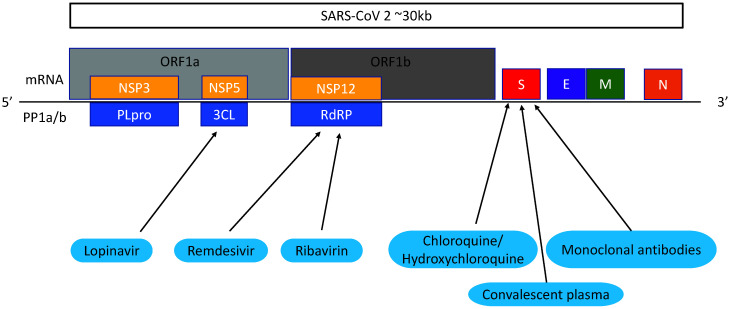
SARS-CoV-2 gene targets. Proteins are translated from the SARS-CoV-2 genome. Lopinavir targets 3CL protease produced by the ORF1a gene. Remdesivir and ribavirin targets RNA-dependent RNA polymerase (RdRp) translated from ORF1b. Chloroquine/ hydroxychloroquine, convalescent plasma and monoclonal antibodies have effects against the spike protein.

Remdesivir (GS-5734) is a broad-spectrum nucleoside analogue which is metabolised by the host cell and converted into nucleoside triphosphate (NTP). NTP targets RNA-dependent RNA polymerase (RdRp) which inhibits the transcription of viral RNA [37] (**Figure 2**). Remdesivir was developed in 2009 by Gilead Sciences [79]. It is most commonly known as a drug to treat Ebola but was proved to be an inferior treatment to other contenders [80].

Remdesivir is currently FDA approved for emergency administration to COVID-19 patients. A study in severe COVID-19 patients showed very positive results. 68% of patients improved and only 13% of patients died [40]. These findings are highly significant, considering the mortality of patients with severe COVID-19 is over 40% [81]. However, viral load was not collected to confirm the antiviral effects and the patient cohort was small and therefore there is a higher chance of false-positive results.

**Figure 3:**
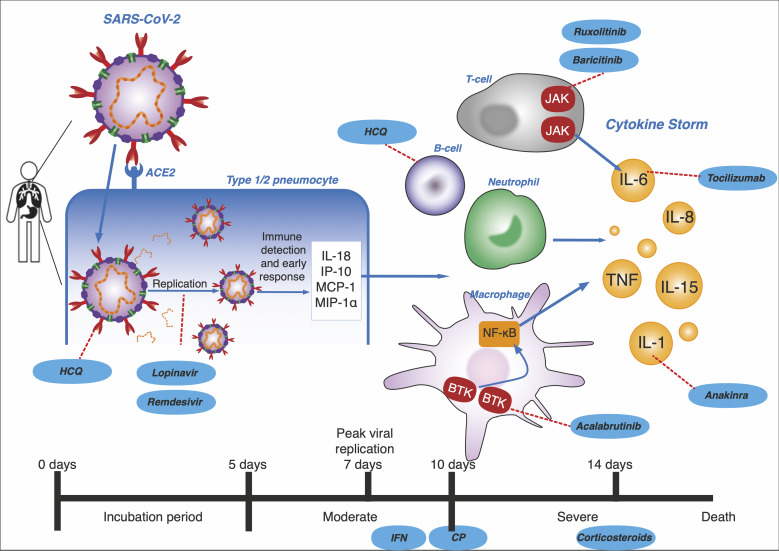
Progression to cytokine storm. Peak viral replication occurs in the initial 7-10 days and primary immune response usually occurs by day 10-14 which is followed by virus clearance [1]. Therefore, therapies appear to be most beneficial when given before 14 days of infection. Days 0-5: incubation period. SARS-CoV-2 enter cell via ACE2 receptors on human type 1/2 pneumocytes and start replicating. Hydroxychloroquine (HCQ) targets glycosylation of viral surface. Lopinavir, remdesivir targets the viral replication. Until day 10: moderate symptoms. The initial immune response produces IL-18, IP-10, MCP-1 and MIP-1α which starts to recruit specific immune cells. Interferon (IFN), HCQ and convalescent plasma (CP) is most effective. Day 10: severe infection leading to risk of mortality. Macrophages via BTK, T-cells via JAK, neutrophils and B-cells produce cytokines, resulting in a cytokine storm by day 14. Ruxolitinib, baricitinib, tocilizumab, anakinra, acalabrutinib and corticosteroids can be used [1].

Remdesivir was studied further in a randomised, double-blinded, placebo-controlled trial of 237 patients in which Wang et al reported a statistically insignificant median recovery time of 21 days compared to 23 days in the control [41]. There was no decrease in mortality rate and more patients receiving the treatment dropped out of the trial due to adverse effects which included gastrointestinal symptoms and worsened cardiopulmonary status. This trial did not complete full enrolment. A randomised, placebo-controlled, double-blinded clinical trial of 1062 patients where patients received the same drug dose as the previous study showed that remdesivir had a superior recovery time of 10 days compared to 15 days in the placebo group [42]. Contrary to Wang, this trial found that compared to the placebo, patients who received remdesivir experienced less adverse effects and the mortality rate decreased from 11.9% to 6.7%. Although this is significant, the high mortality rates reported make it clear that remdesivir alone is unlikely to be sufficient in COVID-19 treatment. This conclusion is supported by the results of a randomised clinical trial which found that despite patients receiving a 5-day course of remdesivir having a significantly better status, it was not clinically important [43]. In accordance with in vivo studies, the drug is most likely only beneficial to patients in the early stages of infection (**Figure 3**).

#### Convalescent plasma

In convalescent plasma (CP) therapy, blood plasma is taken from a patient previously infected by the virus and transfused into a symptomatic patient. The theory is that the antibodies transferred to the symptomatic patient will suppress viremia [1]. Antibodies, or immunoglobins, bind to invading viruses which neutralises the pathogen (**Figure 3**). It can also cause virus destruction by processes like activating the complement pathway or opsonisation via viral Fc receptors. CP is generally regarded as safe and has been recorded to be used for the Spanish Flu pandemic as early as 1918 [44] and has been used to treat Machupo virus, Junin, Lassa fever and Ebola virus [1]. It also reduces mortality rates in patients with severe influenza A [82].

Convalescent plasma therapy is currently FDA approved to be used in COVID-19 patients for investigational purposes. Observational studies have demonstrated it to be safe and patients treated have a significantly improved oxygen saturation, CRP, 30-day survival rate and reduced need for mechanical ventilation [45,46]. Results also suggest CP could alleviate inflammation and overreaction of the immune system. This potential is further demonstrated by a study which found a 76% improvement rate in patients, of which more than half had ARDS [44].

A randomised, open label clinical trial was performed on 103 patients with life threatening COVID-19 [47]. Results show that CP therapy was clearly highly effective in viral clearance. Treatment was associated with 87% negative conversion rate of viral PCR compared to 37.5% of the control group. Although those patients receiving the treatment had a shorter time to clinical improvement and a lower 28-day mortality rate, these results were found to be statistically insignificant. The study was terminated early due to a shortage of patients. Another randomised controlled trial involving 1210 patients found no clinical or mortality improvement between patients receiving CP and patients receiving standard care. There was also no evidence of anti-inflammatory properties [48]. Zeng et al found that treatment inhibits viral shedding but doesn’t reduce mortality, and late treatment intervention could even be attributed to poorer clinical outcomes [49]. In concordance with previous findings in SARS and MERS, the authors recommend transfusion to be given before day 14 of infection.

#### Hydroxychloroquine

Hydroxychloroquine and chloroquine are 4-aminoquinolines. Chloroquine is thought to be a drug that blocks viral entry by inhibiting glycosylation of cell surface receptors, proteolytic processing and endosomal acidification [83]. (**Figure 2**). It also has immunomodulatory effects and inhibits cytokine production, so could potentially help patients with cytokine storm (**Figure 3**). Hydroxychloroquine (HCQ), an analogue of chloroquine, has the same antiviral mechanisms but is associated with less drug-drug interactions and adverse effects [84]. Both drugs are used to treat malaria, lupus and rheumatoid arthritis, and are cheap and easy to attain [83].

Hydroxychloroquine is one of the most controversial treatments and is not recommended by the FDA. The WHO discontinued its trial in July 2020 which followed their temporary suspension of the trial in May after a French hospital found the adverse effects of prolonged QTc heart rate intervals caused death due to sudden cardiac arrest [50]. There was a rapid increase in demand of the drug after USA president Donald Trump publicly claimed to use the drug [85]. Initial trials showed both hydroxychloroquine and chloroquine displayed antiviral effects against SARS-CoV-2 in vitro [86]. Strikingly, an open label, non-randomised hydroxychloroquine clinical trial in France found that patients had clear nasopharyngeal viral results in 3 - 6 days compared to the mean clearance time of 20 days [51].

Other studies show contrasting findings. A randomised, controlled trial of 4716 patients by the Recovery Group found that not only did hydroxychloroquine not provide any benefit, but it also resulted in worse outcomes [52]. The 28-day mortality rate of patients receiving hydroxychloroquine was 27% compared to 25% in those receiving standard care. The group receiving the drug was also associated with longer hospitalization and higher progression to invasive mechanical ventilation. A double-blinded, randomised trial was conducted in patients with severe COVID-19 where half were given low dosages of hydroxychloroquine and half were given high dosages of hydroxychloroquine [53]. Worryingly, patients receiving high dosages had a mortality rate of 39% compared to 15% receiving low dosages. There was also no evidence of viral clearance. Another randomised, double-blind placebo trial showed that the drug did not prevent postexposure prophylaxis for the illness either [54]. In addition to cardiac side effects, hydroxychloroquine has been reported to cause vomiting and abdominal pain in half of the patients treated [55]. A randomised trial of patients with moderate COVID-19 symptoms conducted in China, found that hydroxychloroquine not only failed to provide benefit, but caused a much higher rate of mainly gastrointestinal adverse effects [56]. Hydroxychloroquine is unlikely to be beneficial in the treatment of COVID-19 but it has been suggested that if given at a low dose with an anti-inflammatory drug it might help mitigate the cytokine storm in critically ill patients [84].

### Existing broad-spectrum antiviral drugs

#### Lopinavir and ritonavir

Based on clinical evidence in SARS-CoV and MERS-CoV, the most commonly used antivirals for COVID-19 are lopinavir, ritonavir and ribavirin. Lopinavir/ritonavir (LPVr) combined formulates Kaletra, an FDA approved drug used to treat HIV. Lopinavir inhibits 3-chymotrypsin-like protease (3CL^pro^) which disrupts viral replication and RNA release from host cells [87] **(****Figure 2****).** Ritonavir is a human CYP3A4 enzyme inhibitor. In addition, lopinavir efficacy was found to be inferior to remdesivir and interferon-β [88]. Randomised, controlled clinical trials of COVID-19 patients found that LPVr was not associated with clinical improvement [57,58]. Furthermore, up to 50% of patients could experience adverse effects including gastrointestinal problems and hepatoxicity [57].

#### Interferon

Interferons (IFNs) are cytokines discovered to have antiviral properties in 1957 and have since been approved for clinical use by the FDA [89]. They are widely available to treat hepatitis, leukaemia and carcinoma [90]. Human cells naturally produce type I interferons, IFN-α and IFN-β, in response to viral infections. Of these, IFN-β is the most potent [90]. They are crucial in inhibiting viral replication and can also activate the transcription of hundreds of genes which produce virus controlling proteins [91] (**Figure 3**).

IFN-β 1a combined with Lopinavir/Ritonavir was previously approved for international trialling by WHO. A randomised clinical trial of 81 patients found that IFN-β 1a decreased the 28-day mortality rate from 43.6% to 19%, although it did not change time taken to reach clinical response [59]. Further support of this treatment was found in a recent randomised, double-blind, placebo-controlled trial which found patients receiving nebulised IFN-β 1a were twice as likely to recover than the standard group (44% vs 22%) [60]. Similarly, IFN-β 1b is also thought to be beneficial. The results of a randomised clinical trial in 66 patients showed that it caused a significantly shorter time for clinical improvement and it reduced 28-day mortality from 18.8% to 6% [61]. However, an open-label, randomised, phase 2 clinical trial carried out in 127 patients suggested that although patients with moderate symptoms had a significantly better clinical and virologic response, it is unlikely to benefit patients with severe symptoms [62].

#### Corticosteroids

Corticosteroids are hormones naturally produced by the adrenal cortex, principally in the form of glucocorticoids and mineralocorticoids. They have widespread effects on the human body including metabolic regulation and reducing vasodilation [92]. Glucocorticoids bind to glucocorticoid receptors (GR) on the cell cytoplasm and regulates gene expression [93]. This can produce anti-inflammatory and immunosuppressant factors which is useful pharmacologically. Synthetic glucocorticoids include betamethasone which treats asthma, and prednisolone which treats COPD and rheumatoid arthritis.

Due to its controversy, corticosteroids were not previously recommended by WHO to treat COVID-19. However, on 2 September 2020 it was approved for the treatment of patients in critical condition [94]. Theoretically, corticosteroids should inhibit the symptoms of a “cytokine storm” which only presents in patients classified as severe [63,64,95] (**Figure 3**). Clinical studies reported that around 20%-50% of observed COVID-19 patients received this treatment. In an observational study, Zhang et al suggested that corticosteroids could possibly produce early clinical benefits in COVID-19 [36]. The effectiveness of corticosteroid therapy was contested by Zhou et al, who thought that high dose corticosteroids could have contributed to poor clinical outcomes in a cohort study [65]. Recent findings have reinstated corticosteroids as an important drug in this pandemic. In contrast to most outcomes of corticosteroid trials [66], the steroid dexamethasone seems very promising. Furthermore, a recent review suggested that hydrocortisones has a similar effectiveness [67]. A trial carried out in hospitalised patients by the Recovery Collaborative Group found dexamethasone reduced the mortality rate for patients on ventilators from 41.4% to 29.3%, and from 26.2% to 23.3% for patients needing oxygen [68]. However, there was no benefit in its use for patients with moderate symptoms. For patients who were not receiving any respiratory support, patients who received dexamethasone had a mortality rate of 17.8%, compared to a 14% mortality in the control group. Prolonged corticosteroid treatment may be effective in saving lives of severe patients who develop ARDS and multiple organ failure, but short therapy is suggested to increase death, so cannot be thought as an effective cure [96].

### Drugs targeting the cytokine storm

The most critically ill COVID-19 patients develop a massive immune response, resulting in a cytokine storm. This causes ARDS and multi-organ failure. Moderation of immune mediators such as Janus Kinase (JAK), bruton tyrosine kinase (BTK) and interleukins (IL) are crucial in saving patient lives (**Figure 3**). JAK inhibitors include baricitinib and ruxolitnib. A clinical trial found patients treated with baricitinib achieved significantly greater clinical improvements. After 2 weeks, 58% of the treated patients were discharged compared to 8% of the control group [69]. It also significantly reduced IL-1β, IL-6, and TNF-α plasma concentrations [70]. Patients given ruxolitinib in a randomised, controlled trial displayed significantly decreased levels of cytokines and numerically faster clinical improvement [71]. Acalabrutinib, a BTK inhibitor, was found to improve symptoms of severe patients [72]. In a cohort study the IL-1 receptor antagonist Anakinra was found to greatly reduce the need for invasive mechanical ventilation and decrease mortality in a cohort study from 73% in the historical group to 25% in the treatment group [73]. This result has been supported by other similar studies [74]. The therapeutic effects of these cytokine storm inhibiting drugs seem favourable, but more high quality, randomised controlled trials are needed to fully assess its affects.

### Development of novel specific treatment agents

#### Monoclonal and polyclonal antibodies

Monoclonal antibodies are produced from immunized animals, antibody human phage libraries and memory B cells of recovered patients [90]. Compared to convalescent plasma treatment, monoclonal antibodies are an alternative strategy which has higher efficacy, is able to be expressed in larger quantities and reduces the risk of antibody dependent enhancement [97].

Tocilizumab is a recombinant, anti-human IL-6R monoclonal antibody that inhibits hyperinflammation in patients with COVID-19. It is currently most well known in treating rheumatoid arthritis, another inflammatory immune disease. A randomised, double-blind, placebo-controlled trial in 389 moderate to severe patients found that tocilizumab improved the combined progression to either mechanical ventilation or death (12% vs 19.3%). However, it did not improve survival alone [75]. Another randomised trial also found that there was no improvement in 28-day mortality in moderate and severe cases [76]. In fact, in more critical cases, tocilizumab may lead to worse outcomes. A randomised, open label trial of severe or critical COVID-19 patients found death at 15 days increased from 3% in the standard care group to 17% in the patients receiving treatment and was linked to a higher occurrence of adverse events [77]. The trial was not completed due to the large number of deaths.

REGN-COV2 is a monoclonal antibody cocktail that target the RBD of the S1 or the S2 segment of spike proteins, thus interfering with entry into the host cell. Recent results of a double- blind, randomised controlled trial of REGN-COV2 show a reduced viral load especially in patient whose immune response had not yet been initiated [78]. Some other potential monoclonal antibodies effective against SARS-CoV-2 include B38, H4 and CR3022 [98,99].

## DISCUSSION

In this rapid review, we summarise the literature on COVID-19 treatments. There is not yet a completely effective cure for COVID-19. This review found no certain evidence of any single therapy to significantly improve clinical outcomes. Of the 40 studies included, 18 reported clinical improvement while 17 reported no significant result and 5 reported a worsening of clinical progression. Most of the studies reporting clinical improvement are observational and have a limited sample size. Many of these studies report that although the therapy studied was effective in improving patient recovery time and symptoms of mild disease, it is not effective in preventing mortality rates of severe patients. In contrast, many randomised controlled trials report no significant improvements or even worsened outcomes.

Timing of administration is crucial in drug efficacy. In accordance with the SARS-CoV-2 peak replication time and the patient’s primary immune response, therapies appear to be most beneficial when given before 14 days of infection [1,49,100,101]. Broad-spectrum antiviral treatments could benefit patients with mild symptoms in the early stages of infection but are ineffective in treating severe disease patients. Remdesivir, convalescent plasma and interferon were found to reduce viral load and improve patient recovery time but failed to prevent mortality. Conversely, dexamethasone and hydrocortisones have been proven to save lives in severe patients but are not effective in mild cases. Other drugs like hydroxychloroquine do not seem to be effective in either mild or severe patients.

There is evidence that remdesivir can improve recovery time in COVID-19 patients and reduce the mortality rate from 11.9% to 6.7% [42]. Nonetheless, other randomised controlled trials suggest that any benefit is not clinically significant [41,43]. These results are consistent with in vitro and animal studies. Remdesivir is highly effective in reducing replication of SARS-CoV-2 in human cell lines [86]. However, in rhesus macaques, although it reduced clinical disease and lung damage, there was no reduction of virus replication in the upper respiratory tract [102]. Current evidence from previous coronaviruses do not suggest added benefit of the drug. In initial studies, remdesivir was found to have prophylactic and therapeutic effects against SARS- and MERS-CoV in human airway epithelial cells and in animals, as well as having antiviral properties against HCoV-299E and HCoV-OC43 [103]. However when used therapeutically in MERS patients, it was unable to prevent mortality or loss of pulmonary function [88].

Convalescent plasma therapy was found to be effective in reducing viral clearance in COVID-19 patients [47,49]. However, there is limited effect on the clinical outcomes of patients [47,48]. Efficacy of CP was initially supported in trials involving SARS and was reported to be effective in decreasing mortality rate in several studies [104]. In deteriorating SARS patients, switching to CP therapy was associated with better outcomes than continuing high dose methylprednisolone [105]. Studies on MERS better reflect the outcomes for COVID-19 patients. Initial experiments on immune camel serum was found to be able to diminish weight loss and lung histological changes, and improve virus clearance in mice [106]. However, it was found that antibody titres collected from recovered patients were too low in the plasma to covey any therapeutic effect [107]. Thus in 2014, the WHO’s stance on CP changed from being the most promising near-term MERS therapy to be only regarded as investigational [90]. In concordance with findings in SARS and MERS, it is recommend transfusion to be given early, before day 14 of infection, for greatest therapeutic effect in COVID-19 patients [49].

A treatment suggesting very limited potential is hydroxychloroquine. The drug was associated with a higher mortality of 27% compared to 25% in the control, as well as longer hospitalisation [52]. There are also several worrying side effects including cardiac symptoms. In previous coronaviruses, chloroquine was shown to inhibit SARS-CoV, MERS-CoV and HCoV-229E in vitro [108], [109]. Hydroxychloroquine was similarly effective in SARS, suggesting it to be a potential pharmacological agent for coronaviruses [84]. However, the effects of chloroquine/ hydroxychloroquine were never fully explored in vivo [110] and the discrepancy with COVID-19 highlights the need for vigorous drug testing upon translation from in vitro studies to patients.

Broad spectrum antivirals LPVr are common treatments for COVID-19 patients. However, it is unlikely to provide any clinical improvement [57,58]. Lopinavir inhibits SARS-CoV-2 in vitro but it was recently found that ritonavir did not [87]. Studies in SARS and MERS provide further evidence of their limitations. Although LPVr inhibited the replication of SARS-CoV, it was not effective in MERS-CoV [111]. Other antivirals such as Ribavirin, a nucleoside analogue that inhibits viral RdRp [112]is similarly unlikely to provide clinical value. In vitro it was found that it could only inhibit SARS-CoV at very high levels which is difficult to achieve clinically [113] and has no benefit in SARS and MERS patients [83]. Although unlikely to be effective alone, broad spectrum antivirals may play a role in conjunction with other therapies.

Several randomised controlled trials demonstrate the benefits of IFN-β. The drug was shown to improve time for clinical improvement and recovery, and also decrease 28-day mortality from 43.6% to 19% [59]. Evidence from previous years show IFN is able to inhibit SARS- and MERS-CoV replication both in vitro and in animals [37] and had maximum effect used in combination with the broad spectrum antivirals, lopinavir/ritonavir and ribavirin [112]. A clinical trial of IFN- α 2a and ribavirin in MERS patients showed improvement in 14-days survival but not 28-day survival. Significantly, IFN-β 1a resulted in a mortality rate of 64% compared to 85% in IFN- α 2a [90].

It is suggested that corticosteroids such as dexamethasone are vital in preventing mortality in patients with severe COVID-19 [68]. Current evidence reported from SARS and MERS suggest varied benefit. A non-randomised and uncontrolled trial showed that 89% of 107 patients who received high dose methylprednisolone recovered from the disease [114]. In contrast, a randomized, double-blinded, placebo-controlled trial showed that corticosteroids caused an increase in patient viral load and decrease in viral clearance [28]. Similarly in MERS, a study concluded that corticosteroids not only had no effect in preventing ARDS and pulmonary fibrosis, it was also associated with osteonecrosis, delirium and aspergillosis [90]. Further contradiction arises in a 2020 randomised trial of ARDS patients which found that methylprednisolone was associated with a significant increase in death [115].

There is promise in new cytokine storm targeting drugs like JAK and BTK inhibitors, but further investigation is required to discern its value for treating critically ill. Previously, monoclonal antibodies were shown to have neutralizing effects in vitro, in mice and in rhesus macaques for SARS and MERS [90,116]. However, there is no evidence that tocilizumab is effective in treating COVID-19 patients and REGN-COV2 requires further investigation to fully understand its effects.

Future prospects of the pandemic could depend mainly on the development of novel therapies like monoclonal antibodies and prevention through effective and widespread vaccination which will be effective against mutated strains. Meanwhile, to halt the spread of the virus, social distancing and good hygiene practises such as handwashing and face coverings or masks are essential [117].

As of 8 March 2021, there are 308 candidate COVID-19 vaccines of which 16 have entered phase 3 trial, and 4 have entered phase 4 [118]. There are several vaccines currently in use. The WHO has issued emergency use listings (EULs) for Pfizer-BioNtech COVID-19 vaccine and AstraZeneca/Oxford COVID-19 vaccine. The FDA have further approved of the Pfizer-BioNtech, Moderna and Janssen vaccines. ChAdOx1 nCoV-19, developed by the University of Oxford and AstraZeneca is in use in the UK. It is a chimpanzee adenovirus-vectored vaccine. Results from phases 1/2 and 2/3 trials show neutralising antibody responses in >99% of participants by 14 days, and potent cellular and humoral immunogenicity in all participants after a second dose [119,120]. Interim results also show up to 71.2% efficacy from the phase 3 Brazil trial [121]. BNT162b2 developed by Pfizer and Biotech, is a mRNA vaccine. It is also being rolled out in the UK, as well as the US and following phase 3 trial is reported to be 95% effective [122]. Prospective studies of ChAdOx1 nCoV-19 and BNT162b2 from real world data in the UK confirms their protective effect against hospitalisation [123,124]. Another mRNA vaccine, mRNA-1273, developed by Moderna is currently rolled out in the US, and is reported to be 94.5% effective on preventing COVID-19 [125].

Other vaccines include the CoronaVac (formerly PiCoVacc) which was found in phase 1 and 2 trials to be safe in inducing neutralizing antibodies with a 97.4% seroconversion rate after 28 days [126]. Other promising vaccines are the adenovirus vectored vaccines. Phase 1 and 2 trials on Ad5 by CanSino Biologics show its safety in causing a 97% antibody seroconversion rate in participants by 28 days [127]. The Janssen vaccine (Ad26) by Johnson& Johnson is similarly favourable in causing neutralising antibodies in over 90% of participants [128].

This review presents a current overview of COVID-19 therapies and links their development to their efficacy in previous coronaviruses for a more comprehensive picture of their likely trajectory. Future directions of the pandemic are considered by discussing initial studies of novel therapies and vaccines. There are also a number of limitations. First, due to the massive amount and fast pace of publications regarding COVID-19, new findings are constantly changing the treatment landscape. We included some preliminary results of ongoing studies in which not all data was available or presented. Second, due to more limited evidence, we were unable to include all identified COVID-19 therapies in this review such as oseltamivir and colchicine. Third, in observational and retrospective studies included, the duration and dose of therapy were not standardised and may be subject to bias. Fourth, the literature was limited to studies published in English and was searched for in only two databases. The search and screening process was not screened twice.

## CONCLUSION

While the pandemic continues to spread worldwide, there is urgent need to understand the benefits and risks of each treatment. Living drug treatment systematic reviews which updates when new evidence becomes available can be followed [129]. Currently there is no single effective treatment against COVID-19. A combination of therapies administered at different stages of infection seems to provide the best outcomes in patients. As most clinical trials are carried out in severe patients, there is a need for more high quality randomised controlled trials, especially of patients in earlier stages of infection. An effective and safe vaccine distributed worldwide will be a turning point in resolving the COVID-19 pandemic.

## Additional material

Online Supplementary Document
