# Supplementary material for: Current COVID-19 treatments: Rapid review of the literature
Source: J Glob Health. 2021 Apr 24;11:10003. doi: 10.7189/jogh.11.10003 (PMC8068411; doi:10.7189/jogh.11.10003)
Supplement: Online Supplementary Document [file jogh-11-10003-s001.pdf]

Supplementary Documents
Table S1- Search Strategies.

Medline	
1	(coronavir* or corona virus* or covid19 or covid 19 or nCoV or novel CoV or novel coronavirus or CoV2 or sarscov2 or sars2 or 2019nCoV or wuhan virus*).mp.
2	Therapeutics/ or (lopinavir or ritonavir or remdesivir or chloroquine or hydroxychloroquine or anakinra or ribavirin or tocilizumab or sarilumab or JAK or barcitinib or corticosteroid* or convalescent plasma or interferon or IFN or monoclonal antibod* or antibodies).mp.
3	1 and 2
4	Limit 3 to (english language and yr=”2019-Current” and (case reports or clinical study or clinical trial or comparative study or multicentre study or observational study or randomised controlled trial or “systematic review”) and medline)
Embase	
1	(coronavir* or corona virus* or covid19 or covid 19 or nCoV or novel CoV or novel coronavirus or CoV2 or sarscov2 or sars2 or 2019nCoV or wuhan virus*).mp.
2	Therapy/ or (lopinavir or ritonavir or remdesivir or chloroquine or hydroxychloroquine or anakinra or ribavirin or tocilizumab or sarilumab or JAK or barcitinib or corticosteroid* or convalescent plasma or interferon or IFN or monoclonal antibod* or antibodies).mp.
3	1 and 2
4	Limit 3 to (english language and yr=”2019-Current” and (clinical trial or randomized clinical trial or controlled clinical trial)and embase)
WHO COVID-19 Database	
1	(tw:(coronavir* OR covid19 OR covid 19 OR nCoV OR "novel CoV" OR "novel coronavirus" OR CoV2 OR sarscov2 OR sars2 OR 2019nCoV OR "wuhan virus")) AND (tw:(Therapy)) AND (tw:(lopinavir or ritonavir or remdesivir or chloroquine or hydroxychloroquine or anakinra or ribavirin or tocilizumab or sarilumab or JAK or baricitnib or corticosteroid* or convalescent plasma or interferon or IFN or monoclonal antibod* or antibodies)) AND (PT: "clinical trial" OR "systematic review")
